# Assessing environmental impacts of offshore wind farms: lessons learned and recommendations for the future

**DOI:** 10.1186/2046-9063-10-8

**Published:** 2014-09-14

**Authors:** Helen Bailey, Kate L Brookes, Paul M Thompson

**Affiliations:** 1 Chesapeake Biological Laboratory, University of Maryland Center for Environmental Science, 146 Williams Street, Solomons, MD 20688, USA; 2 Marine Scotland Science, 375 Victoria Road, Aberdeen AB11 9DB, UK; 3 Institute of Biological and Environmental Sciences, Lighthouse Field Station, University of Aberdeen, George Street, Cromarty, Ross-shire IV11 8YJ, UK

**Keywords:** Marine mammals, Seabirds, Wind turbine, Underwater noise, Collision risk, Human impacts, Cumulative impact assessment, Population consequences

## Abstract

Offshore wind power provides a valuable source of renewable energy that can help reduce carbon emissions. Technological advances are allowing higher capacity turbines to be installed and in deeper water, but there is still much that is unknown about the effects on the environment. Here we describe the lessons learned based on the recent literature and our experience with assessing impacts of offshore wind developments on marine mammals and seabirds, and make recommendations for future monitoring and assessment as interest in offshore wind energy grows around the world. The four key lessons learned that we discuss are: 1) Identifying the area over which biological effects may occur to inform baseline data collection and determining the connectivity between key populations and proposed wind energy sites, 2) The need to put impacts into a population level context to determine whether they are biologically significant, 3) Measuring responses to wind farm construction and operation to determine disturbance effects and avoidance responses, and 4) Learn from other industries to inform risk assessments and the effectiveness of mitigation measures. As the number and size of offshore wind developments increases, there will be a growing need to consider the population level consequences and cumulative impacts of these activities on marine species. Strategically targeted data collection and modeling aimed at answering questions for the consenting process will also allow regulators to make decisions based on the best available information, and achieve a balance between climate change targets and environmental legislation.

## Introduction

**Figure 1:**
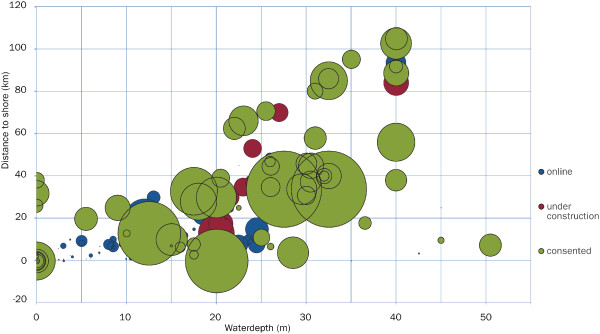
**Average water depth and distance to shore of offshore wind farms (reproduced from ref. 3, source EWEA).** Operational (online), under construction and consented wind farms in Europe up to the end of 2013 are occurring at increasing water depths and distances from shore. The circle size represents the total power capacity of the wind farm.

Efforts to reduce carbon emissions and increase production from renewable energy sources have led to rapid growth in offshore wind energy generation, particularly in northern European waters [1,2]. The first commercial scale offshore wind farm, Horns Rev 1 (160 MW with 80 turbines of 2 MW), became operational in 2002. The average capacity of turbines and size of offshore wind farms have been increasing since then, and they are being installed in deeper waters further from the coast. By the end of 2013, operational wind farms were in an average water depth of 16 m and 29 km from shore in Europe [3] (Figure 1). With technological advances in the future [4] there is likely to be a continued increase in the size of offshore wind projects [3], but there are still uncertainties about the effects on the environment [5]. The novelty of the technology and construction processes make it difficult to identify all of the stressors on marine species and to estimate the effect of these activities [6].

The major environmental concerns related to offshore wind developments are increased noise levels, risk of collisions, changes to benthic and pelagic habitats, alterations to food webs, and pollution from increased vessel traffic or release of contaminants from seabed sediments. There are several reviews of the potential impacts of offshore wind energy on marine species e.g. [5-7]. As well as potential adverse impacts, there are possible environmental benefits. For example, wind turbine foundations may act as artificial reefs, providing a surface to which animals attach. Consequently there can be increases in the number of shellfish, and the animals that feed on them, including fish and marine mammals [8-11]. A second possible benefit is the sheltering effect. A safety buffer zone surrounding the wind turbines may become a de-facto marine reserve, as the exclusion of boats within this zone would reduce disturbance from shipping. Exclusion of some or all types of fishing could also result in local increases in prey abundance for top predators, whilst reducing the risk of bycatch in fishing gear [9]. Further research is required to understand the ability of wind turbines to attract marine species and the effect of excluding fisheries. Finally, there may also be opportunities in the future to combine offshore wind farms with open ocean aquaculture [12].

**Figure 2:**
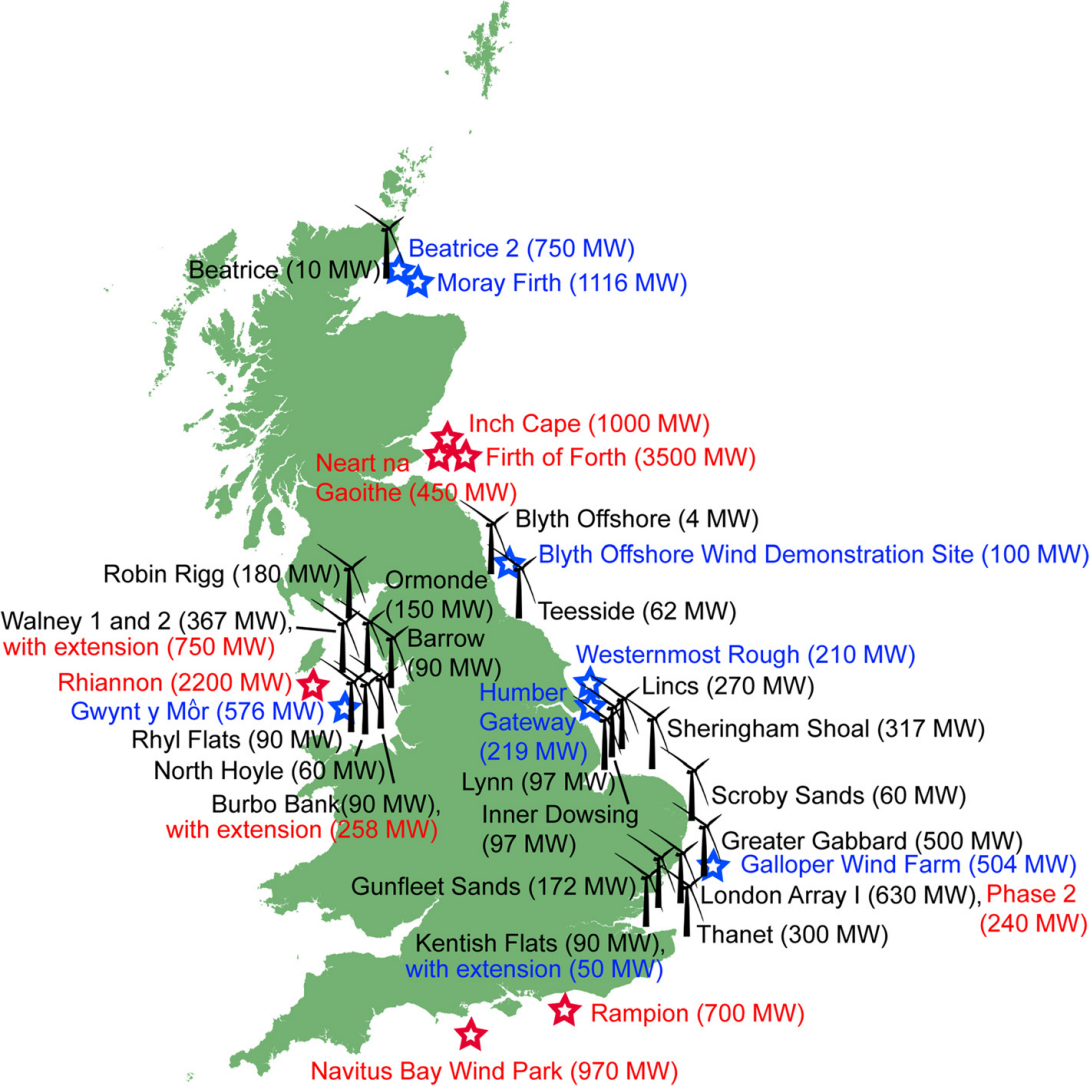
**Offshore wind farms around the U.K., July 2014.** This includes wind farms in operation (black wind turbines), those consented and under development (blue stars), and in the proposal and planning stages (red stars).

Over 2,000 wind turbines are installed in 69 offshore wind farms across Europe, with the greatest installed capacity currently in the U.K. (Figure 2) [3]. As the number of offshore wind farms has increased, approaches for environmental monitoring and assessment have improved over time. However, there are still few studies that have measured the responses of marine species to offshore wind farm construction and operation, and none have yet assessed longer term impacts at the population level. In Europe, legislation requires consideration of cumulative impacts, defined as impacts that result from incremental changes caused by other past, present or foreseeable actions together with the project [13]. However, approaches for cumulative impact assessments currently vary in terms of their transparency, efficiency and complexity, and this is an active area of research development [14]. In addition to assessing and measuring impacts, it is also necessary to develop decision support tools that will assist regulators with determining whether a proposed development can be legally consented.

In this paper, we first briefly review the potential impacts of offshore wind developments on marine species. We then identify the key lessons that have been learned from our own studies and others in Europe, primarily focusing on marine mammals and seabirds. Much of the environmental research that has been conducted in relation to offshore wind energy has concerned the impact of sound exposure for marine mammals and the risk of collisions with turbines for seabirds. We identify where knowledge gaps exist that could help to improve current models and impact assessments. Finally, we discuss emerging technologies and make recommendations for future research to support regulators, developers and researchers involved in proposed developments, particularly in countries where the implementation of offshore wind energy is still in its early stages.

### Impact pathways

The potential effects of offshore wind farm construction and operation will differ among species, depending on their likelihood of interaction with the structures and cables, sensitivities, and avoidance responses. Studies have generally focused on marine mammals and seabirds because of stakeholder concerns and legal protection for these species and their habitats. The construction phase is likely to have the greatest impact on marine mammals and the activities of greatest concern are pile driving and increased vessel traffic [15]. Pile driving is currently the most common method used to secure the turbine foundation to the seafloor, although other foundation types are being developed [4]. The loud sounds emitted during pile driving could potentially cause hearing damage, masking of calls or spatial displacement as animals move out of the area to avoid the noise [16,17]. Fish could similarly be affected by these sounds [17-20]. There is also a risk to marine mammals, sea turtles and fish of collision and disturbance from vessel movements associated with surveying and installation activities.

During operation of the wind turbines, underwater sound levels are unlikely to reach dangerous levels or mask acoustic communication of marine mammals [21,22]. However, this phase of the development is of greatest concern for seabirds. Mortality can be caused by collision with the moving turbine blades, and avoidance responses may result in displacement from key habitat or increase energetic costs [23,24]. This may affect birds migrating through the area as well as those that breed or forage in the vicinity.

During operation, cables transmitting the produced electricity will also emit electromagnetic fields. This could affect the movements and navigation of species that are sensitive to electro- or magnetic fields, which includes fish species, particularly elasmobranchs and some teleost fish and decapod crustaceans, and sea turtles [25-27]. Commercial fish species may potentially be positively affected if fishing is prohibited in the vicinity of the wind farm, although this could result in a displacement of fisheries effort and consequent change in catches and bycatch.

The specific species of greatest concern will differ among regions depending on their occurrence and protection status. For example, assessments of impacts upon marine mammals in Europe have generally focused on small cetaceans (particularly harbor porpoises (*Phocoena phocoena*)) and pinnipeds (primarily harbor seals (*Phoca vitulina*)). These species are common in such areas and the EU Habitats Directive (92/43/EEC) requires governments to establish Special Areas of Conservation for their protection. However, in other locations, marine mammal species listed under the Endangered Species Act, such as the North Atlantic right whale (*Eubalaena glacialis*), blue whale (*Balaenoptera musculus*), humpback whale (*Megaptera novaeangliae*), and fin whale (*Balaenoptera physalus*), may be of greater concern. Based on their call frequencies, these large whales are considered to be sensitive to the low frequency sounds produced during pile driving [16,28,29].

There is also a paucity of information on the effects of human-generated sound on fish [18,20,30,31]. Evidence of injury from pile driving sounds in a laboratory simulated environment has been reported for several fish species [32-34]. Recovery tended to occur within 10 days of exposure and is unlikely to have affected the survival of the exposed animals. Common sole larvae (*Solea solea*) also survived high levels of pile-driving sound in controlled exposure experiments [35]. However, a behavioral response was triggered in cod (*Gadus morhua*) and sole by playbacks of pile driving sounds in the field and was initiated at a much lower received sound level [36]. This could consequently result in a large zone of behavioral response. The sounds produced by offshore wind farms may also mask fish communication and orientation signals [30]. These responses need to be investigated further to determine their potential effect on foraging, breeding and migration, and require the ability to record the movements of fish as well as the measurement of sound pressure levels and particle motion since fish are sensitive to both [18,37]. Some fish species are short-lived and highly fecund reducing the likelihood of any longer-term population level effects from wind farm noise and disturbance. However, this is not true of all fish species and there are endangered species, such as the Atlantic sturgeon (*Acipenser oxyrinchus*), those listed as vulnerable, such as the basking shark (*Cetorhinus maximus*), and potential impacts to fisheries which may need to be taken into consideration.

Much of the early work investigating impacts upon bird populations at European sites has focused on species of migratory or wintering waterfowl [23,38]. There is much less known about potential collision risk or displacement for the broader suite of seabird species that occur in many of the areas currently being considered for large scale wind farm developments. Migrating bats have also been found to occur offshore [39,40], although relatively little research on their offshore distribution, collision risk and potential displacement by offshore wind farms has been done compared to that for wind farms on land [39,41].

Other taxonomic groups such as sea turtles are rare visitors to coastal European waters, and have not been considered at high risk from the effects of offshore wind farms. However, in other areas, for example along the North American coast, there may be sea turtle nesting or breeding grounds in the vicinity of proposed sites [42]. It has recently been determined that the hearing sensitivity of leatherback turtles overlaps with the frequencies and source levels produced by many anthropogenic sounds, including pile-driving [43]. This highlights the need for a better understanding of the potential physiological and behavioral impacts on sea turtles.

### Lessons learned

**Figure 3:**
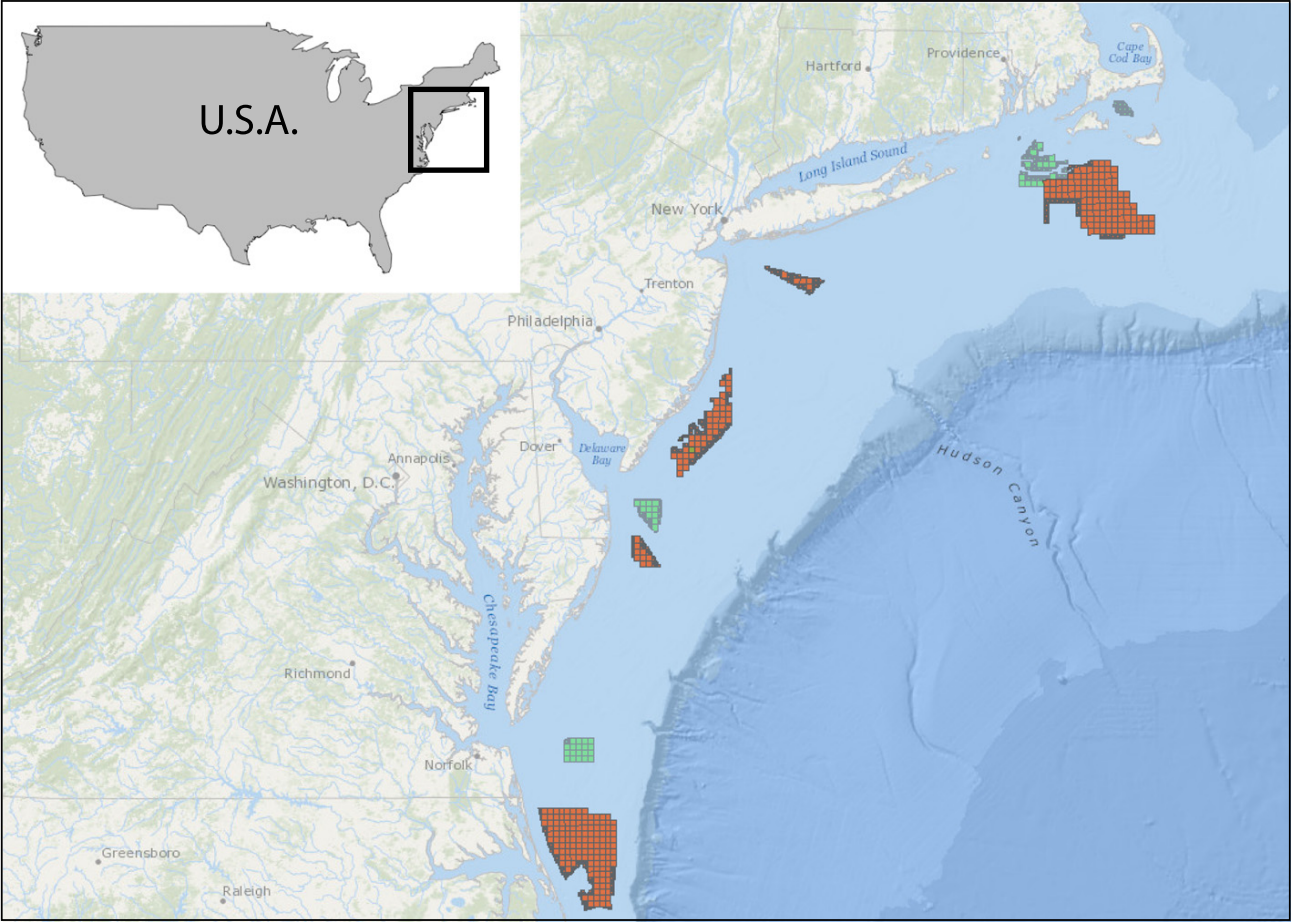
**Potential wind energy areas in the Mid-Atlantic off the U.S.A.(source BOEM).** There are no commercial wind farms currently on the Atlantic outer continental shelf off the U.S.A., but the Bureau of Ocean Energy Management (BOEM) has designated wind energy areas, which are in the planning (orange squares) and leased (green squares) stages (July 2014).

Environmental research for offshore wind energy has evolved over time in Europe as a better understanding of the type of information and analysis that best informs decisions about the siting of offshore wind facilities has been developed. Other countries interested in offshore wind energy, such as the U.S.A. (Figure 3), may therefore benefit from the European experience and hindsight to maximize the potential success of their projects [44]. Based on our experiences relating to marine mammals and seabirds, the key lessons learned that we have identified are:

1. Define the area of potential effect

● Identifying the area over which biological effects may occur to inform baseline data collection.

● Determining the connectivity between key populations and proposed wind energy sites.

2. Identify the scale and significance of population level impacts

● The need to define populations, identify which populations occur within the wind energy site and the area of potential effect, and their current status.

● The requirement for demographic data and information on vital rates to link individual responses to population level consequences.

● Research to test and validate modeling assumptions and parameters.

3. Validate models through measuring responses in the field

● Use of a gradient design to determine the extent of spatial displacement as a result of offshore wind energy development and how this may change over time.

● Utilization of techniques with the power to detect changes.

● Coordination of human activities and monitoring in the vicinity of wind energy sites.

4. Learn from other industries to inform risk assessments and the effectiveness of mitigation measures.

● Onshore wind energy, seismic surveys and floating oil platforms.

We will discuss each of these lessons learned in more detail and then provide recommendations for further research to fill identified knowledge gaps, test existing models and improve future environmental impact assessments (EIA).

### The area of potential effect

To evaluate the impact of a proposed activity on marine species, it is necessary to have sufficient baseline information on distribution, abundance and their trends within the area of potential effect. This is particularly challenging for many marine species since some stressors, such as underwater sound, can travel long distances, and these species are often highly mobile and/or migratory. Consequently, the area of potential effect can extend far beyond the immediate vicinity of the proposed development. For example, the sound produced during pile driving may travel tens of kilometers underwater, which could cause behavioral disturbance to marine mammals and fish over a large area [17]. Early baseline studies in relation to marine mammals and the impact upon them from underwater pile driving noise were designed on relatively uncertain estimates of the area of potential effect, and some of the control sites used were subsequently identified as being impacted [45,46].

Pile-driving sounds were recorded to determine the received levels at a range of distances during the construction of the Beatrice Demonstrator Wind Farm project in the Moray Firth, Scotland [47] (Figure 2). This wind farm consisted of two 5 MW turbines in water 42 m deep with 1.8 m diameter tubular steel piles to secure the jacket foundation structure to the seafloor. Pile-driving sounds were recorded at 0.1 to 70 km away and the peak to peak sound pressure levels ranged from 122–205 dB re 1 μPa. Based on the measurements within 1 km, the source level was back-calculated and estimated to be 226 dB re 1 μPa at 1 m [47]. Each pile required thousands of hammer blows and was struck about once every second.

Since pile-driving involves multiple strikes, it is considered a multiple pulse sound. The sound exposure level (SEL) is a measure of the energy of a sound and depends on both the pressure level and duration [28]. This can be summed over multiple strikes to give the cumulative SEL [19,28]. The cumulative energy level over the full pile-driving duration gives a measure of the dose of exposure, assuming no recovery of hearing between repeated strikes, and is necessary for assessing cumulative impacts.

There were, and still are, insufficient data available to develop noise exposure criteria for behavioral responses of marine mammals to multiple-pulsed noise such as pile driving [28]. Evidence of behavioral disturbance from sounds arising from pile-driving has been obtained through simulated, playback, and live conditions, and indicates that the zone of responsiveness for harbor porpoises may extend to 20 km or more [45,46,48-52]. However, response distances will vary depending on the activity being undertaken by the animal when it is exposed to the sound, the sound source level, sound propagation, and ambient noise levels [16,28,53]. Limited understanding of the role of these different environmental, physical and biological factors currently constrain assessments of the potential scale of impact at particular development sites.

Collecting baseline information for such large areas of potential effect presents a number of challenges. In cases where there is little or no existing information on the species of concern, such as their distribution and abundance in more offshore areas, it can be difficult to determine appropriate designs for impact studies [49]. The logistical difficulties of working offshore, along with financial limitations, may additionally restrict the number of sampling sites and replicates. It has been recommended that at least two years of baseline data are necessary for a sufficient description of species occurrences [54]. However, whilst this may provide information on seasonal variability, longer time-series of data are ideally required to capture inter-annual variability in order to identify the effects of construction activities over natural variation (which may be high) [54,55]. Given that data collection over these large spatial and temporal scales will be so difficult to achieve, it is crucial that studies are targeted to focus upon those data which are critical for supporting decision making. Baseline data and modeling should be targeted to answer specific questions relating to the consenting process, meaning that monitoring and site characterization requirements may differ under different legislative systems. For example, consent may require an understanding of the connectivity of a proposed wind energy site with key protected populations. In Scottish waters, this requirement has focused research efforts on areas within and surrounding proposed wind energy sites, and between these sites and EU designated Special Areas of Conservation (SACs) for harbor seals and for bottlenose dolphins [56-58].

For birds, the operational phase of wind farms is likely to present the biggest risk. Vulnerability and mortality at onshore wind turbines has been identified as being related to a combination of site-specific, species-specific and seasonal factors [59]. The development of collision risk models for seabirds requires information on their spatial distribution and flight heights to determine the likelihood of co-occurrence with the wind turbine blades, and their avoidance response to estimate the mortality risk [60]. However, much of this relies on expert-based estimates because there are very few empirical data on flight heights for different seabird species [24]. Although there have been estimates of flight heights during ship-based surveys where they are classified into altitude categories there can be large inter-observer differences [61]. One recent approach to address this data gap is to model flight height distributions based on compilations of survey data [62,63].

While information on site-specific flight heights of bird species is lacking, there is even less information on avoidance responses to large offshore wind farms by birds. The few studies examining avoidance behavior involved tracking eider ducks (*Somateria mollissima*) and geese by radar. These studies documented a substantial avoidance response by these migrating birds, which reduced the collision risk [23,64]. There is a need for empirical data on both broad and fine-scale avoidance responses to improve the reliability of predictions from collision risk models [65]. There should also be a focus not only on estimates of mortality, but also of the energetic consequences of avoidance and displacement behaviors [66], and their impacts on survival and fecundity. The cumulative impacts of different disturbance activities (such as ship and helicopter traffic) and multiple wind energy sites within the migration pathway or home range of a population should also be considered [67].

### Population level impacts

The regulatory requirements for assessing the impacts of a proposed activity and determining whether it is biologically significant will vary among countries. However, in general this process will require populations to be defined, identifying which of these populations occur within the area of potential effect, and understanding their current status to determine whether the impact will be significant. The complexity of approaches, models and simulation tools to support these assessments has greatly increased over time [24,58,68]. However, there are still many knowledge gaps concerning behavioral responses, particularly on the consequences of any behavioral change on vital rates. For example, there is a growing understanding that anthropogenic noise, such as pile-driving, may affect the behavior of marine mammals and lead to spatial displacement [69]. However, there have been no empirical studies linking the consequences of this behavioral response to longer term population change. Similarly, there are concerns that the presence of wind farms may displace seabirds from preferred foraging areas [24], but there is limited understanding either of the extent of such effects or of the individual and population consequences of displacement, should it occur.

For other management issues, such as bycatch, estimates of Potential Biological Removal have provided management limits for human-caused mortality in mammals [70,71], but this approach cannot be used for assessing non-lethal impacts. To address this, a framework was developed called “Population Consequences of Acoustic Disturbance” (PCAD) [29,72]. The aim of this approach is to link behavioral responses by individuals and their vital rates to determine the consequences for the population. In addition to the spatially-explicit information on distribution and abundance typically collected for impact assessments, this approach also requires knowledge of dose–response relationships to link behavioral responses and demographic parameters. Since a general characterization of the dose–response relationship between received noise levels and changes in vital rates does not exist for marine mammals, expert judgment has been used to link individual impacts to changes in survival or reproductive rates [73].

Given the uncertainties involved, the population level assessments required from developers by U.K. regulators have been very conservative, and are expected to overestimate the impacts to populations. Nevertheless, the application of such an approach to a harbor seal population suggested that the population trends were largely driven by the baseline dynamics of the population and, even in a worst-case scenario of impacts, only a short term reduction in numbers would be expected to occur [58]. The long-term dynamics appeared relatively robust to uncertainty in key assumptions, but there is still a strong reliance on expert judgment and many assumptions are made. Focused studies around subsequent developments are now required to test these modeling assumptions and frameworks to ensure they are robust and, if appropriate, made less conservative in the future.

There is also a strong reliance on expert judgment in seabird collision risk models and sensitivity indices [24,60]. Avoidance rates are applied to collision risk models, but for many species they are not based on empirical data. Work is ongoing to provide estimates of these, but has been hampered by a lack of suitable techniques. The importance of this human-induced mortality on seabirds may depend on the current status of the population, with conservation concerns potentially being greater for populations that are currently in decline. For example, black-legged kittiwakes (*Rissa tridactyla*) have declined by more than 50% since 1990 in the North Sea [74]. The cause of this decline has mainly been attributed to poor breeding success as a result of reduced recruitment of their prey species, the lesser sandeel (*Ammodytes marinus*), linked to warm winters and the presence of a local sandeel fishery [74]. Black-legged kittiwakes generally fly below the minimum height of any turbine’s rotor blades, but there were approximately 15.7% of flights that occurred within a generic collision risk height band defined as 20–150 m above sea level [62] and their avoidance response is unknown. The potential additional mortality that offshore wind farms could induce for this declining population makes this species of particular concern in the environmental assessment and consenting process.

Seabirds are considered at their most vulnerable when wind energy sites are proposed near their breeding colonies. During the breeding season, they make regular trips between their nest and foraging grounds. This could reduce the collision risk for wind farms proposed further offshore, but there is generally less known about the distribution and habitat use of seabirds in these areas outside of the breeding season, and their connectivity with any protected areas. As wind farms move further offshore such knowledge gaps will need to addressed.

### Measuring responses

A BACI (before-after-control-impact) design was initially recommended to assess the responses of marine mammals to wind farm construction and operation [54]. However, this type of design is limited in its ability to characterize spatial variability, assigning samples to only a treatment or control strata [75]. There are also arbitrary requirements for the selection of control sites, which include being far enough away to be unaffected by the potential disturbance, but close enough that the areas are comparable. Some stressors have a large area of potential effect, which makes it difficult to identify suitable control sites with similar ecological characteristics. Differences in variability between sites can also be a problem in statistically detecting impacts [76]. The BACI design is appropriate where there are defined boundaries for the impacted areas, but a gradient design will be more sensitive to change when a contaminant or sound disperses with distance from a point source. A gradient design requires classifying samples according to distance and removes the issue of selecting a control site. It is also more powerful than a randomized Control Impact design at detecting changes due to disturbance [77]. It has recently been demonstrated to be more effective in terms of studying spatial displacement of harbor porpoises in response to pile-driving and detecting how temporal effects differ with distance [48,78]. Furthermore, whilst BACI designs provide opportunities to identify whether or not impacts have occurred, gradient designs can also be used to assess the spatial scale of any impacts, thus informing future spatial planning decisions.

Data collection techniques used for characterizing a site in the planning stages may not be the most appropriate tools for assessing impacts. Visual surveys for both birds and mammals have generally been used to describe their abundance and distribution in planning applications. These techniques are unlikely to have enough power to detect changes in behavior or fine scale spatial or temporal shifts in distribution, since observers can only be in one place at a time and can only reliably survey in calm sea conditions during daylight hours. Our research has shown that acoustic methods for assessing impacts to marine mammals have much greater power to detect change [49,55], and techniques such as GPS tracking, radar, and fixed cameras are likely to provide more useful data for seabirds [64]. GPS tracking has been used for many species and provides high resolution data. In a recent study, it revealed harbor seals foraging around wind turbines in the North Sea [11]. Acoustic telemetry has been a valuable tool for tracking fish [79] and also turtle movements [80], and the technology now exists to use this to examine the long-term, fine-scale movements of aquatic animals [81].

The presence of other disturbance sources unrelated to the wind farm activity may compromise efforts to compare periods before and after construction events. For example, during our study of the impacts of the Beatrice Demonstrator Wind Farm project, we later discovered that hydrographic and seismic surveys had also been conducted in the area during the construction period [49]. Determining the cause of any observed effects is therefore confounded by these additional activities. Communication and coordination amongst planners, regulators, industry and scientists is therefore essential to ensure that impact studies can be properly designed, and any cumulative impacts caused by multiple events during construction or by other human activities can be taken into account [14,82]. Greater involvement of species group specialists during the planning process and engineering design phases may also help to minimize any environmental conflicts at an early stage. Careful spatial planning of wind farms has been identified as a key factor for profitability and environmental protection [83]. Consideration of the increased development of local ports to support construction and maintenance of offshore wind farms and the consequent environmental impacts is also important.

### Learning from other industries

There are three existing industries whose experiences have been usefully applied to environmental research surrounding offshore wind energy developments in European waters. These are onshore wind farms, seismic surveys for oil and gas exploration, and floating oil platforms. Our knowledge of bird vulnerability and mortality from wind farms has largely been based on those on land. Direct measurements of mortality from offshore wind farms are much more difficult because of the difficulty of finding corpses at sea. The lack of direct measurements of flight height distributions and avoidance responses for many seabird species means there is still considerable uncertainty in the mortality estimates and the consequent energetic costs of avoidance behaviors for offshore wind farms, but the modeling approaches developed for terrestrial wind farms have provided a robust framework to begin these assessments [84,85].

Airguns used in seismic surveys for oil and gas exploration produce loud multiple pulsed noises with energy mainly below 1 kHz, but also extending to much higher frequencies [55,86]. Pile-driving also produces loud multiple-pulsed sounds, although they tend to be more broadband with the major amplitude at 100–500 Hz compared to a seismic airgun array at 10–120 Hz [87]. In addition, pile-driving has a shorter interval between pulses at about one second [47,88] as opposed to seismic airgun surveys at 10 seconds or more [87]. Changes in distribution and vocal behavior by marine mammals [55,89-92], and diving behavior by loggerhead turtles [93] have been observed to occur in response to seismic surveys. Following environmental concerns about the impact of these explosive sounds, underwater sound propagation models have been developed to estimate received levels. These are used to determine the distance at which injury or disturbance may occur and to develop mitigation and monitoring plans to reduce noise exposure [94,95]. These approaches have often been adopted during assessments and mitigation of pile-driving activity at offshore wind farms.

Mitigation measures for marine mammals during seismic surveys typically include a soft start or ramp-up to gradually increase the intensity of an airgun array up to full power over a period of 20 minutes or more. This approach is to allow sufficient time for animals to leave the immediate vicinity and avoid harmful noise levels. Similar approaches have been applied to the blow energy intensity during pile-driving. However, whilst this mitigation measure is implemented as a ‘common sense’ approach, no studies have yet investigated its effectiveness systematically [94]. The form, probability and extent of a marine mammal’s response to anthropogenic sound will be affected by a variety of factors. Animals may have different tolerances for increasing sound levels depending on their current behavior, experience, motivation and conditioning [53]. One study observed an avoidance response away from the ramp-up of a 2-D seismic survey by a subgroup of pilot whales (*Globicephala macrorhynchus*) that began when they were 750 m from the airgun array [96], but interpreting the reactions of animals can be difficult because responses can be vertical and/or horizontal. There is a need for further research to assess the efficacy of the ramp-up soft start procedure for mitigating effects on marine mammals.

Another mitigation measure typically used is the monitoring of an exclusion zone. Marine mammal observers are required to visually, and sometimes also acoustically, monitor within a zone in close proximity to the source to ensure the absence of marine mammals (and possibly other protected species such as sea turtles) before beginning piling e.g. [97]. This zone may be a pre-defined fixed distance from the source or based on the expected sound levels. However, there is generally a mismatch between the relatively small area monitored for animals and the potential area of impact, which is likely to be considerably larger [98]. The exclusion zone is aimed at reducing near-field noise exposure and protecting animals from direct physical harm.

Visual observations will be limited during poor visibility conditions and for deep-diving species, such as beaked whales. It is also recognized that this is unlikely to be effective in mitigating behavioral responses over greater distances and that disturbance in the far-field is still likely to occur [55,86,99]. The use of real-time technologies, such as passive acoustic monitoring [100], may be a cost-effective approach to achieve detection coverage over a much larger area for vocalizing animals. Detailed studies to estimate received levels at various distances should be conducted during the planning stages to take into account variations in sound propagation among locations and use this, together with spatiotemporal information on marine mammal occurrence, to identify priority areas for monitoring and mitigation. Current mitigation plans also do not consider the impacts on marine mammal prey species. It should be identified whether any prey species (e.g. fish, squid) are potentially sensitive to noise and disturbance and considered in management plans accordingly to avoid secondary, trophic-level effects, as well as impacts to the fishing industry [18,98]. Efforts are underway to develop technologies to reduce source levels and noise propagation around offshore wind farm sites to help minimize biological impacts e.g. [101].

One measure that could reduce or eliminate the need for pile-driving is the development of floating wind turbine technologies, which are now being considered for deep water (>50 m) sites [2,4,102]. Concerns have been raised over possible entanglement risk in the moorings used to secure the platform to anchors on the seabed. However, the risk would appear to be small as the cables will be under tension and such moorings would be very similar to those widely used for floating oil platforms. Assessments of interactions with wildlife and existing floating oil platforms could therefore inform risk assessments for floating offshore wind turbines and identify what species or groups, if any, may be vulnerable to entanglement.

### The future

#### Emerging technologies

**Figure 4:**
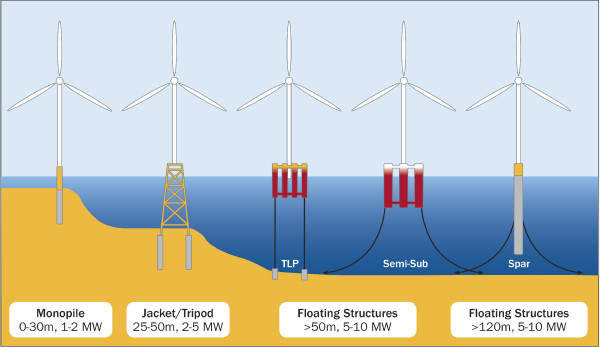
**Types of offshore wind turbine foundations (reproduced from ref. 102, source Principle Power).** Monopile and tripod/jacket foundations are currently proven technologies. Floating structures have been using three main types of foundations, which are adapted from the oil and gas industry: the Tension Leg Platform (TLP), semi-submersible (Semi-sub), and Spar Buoy (Spar).

The greatest change that is likely to occur in offshore wind energy is the increased use of floating foundations. These are designed for deep water areas where the water depth is greater than 50 m (Figure 4). They can currently be used in water depths up to about 300 m but have the potential to reach water depths of up to 700 m, which would greatly increase the potential area for offshore wind energy development [4]. There are many possible designs for floating wind turbines and much more research needs to be done to determine the feasibility of these different options [2]. The first floating wind turbine was installed off Norway in water 220 m deep [4]. Experimental floating turbines have also been installed off Sweden and Portugal, with the latter being a full-scale 2 MW grid connected model [103]. A floating turbine demonstration project of 2 MW off Japan is being followed by a plan for a 1 GW wind farm consisting of up to 143 floating turbines scheduled for start-up in 2018 [104]. There are also currently proposals in the planning system for floating wind turbines off Scotland (http://www.scotland.gov.uk/Topics/marine/Licensing/marine/scoping). The difference in construction of these floating foundations from those that are fixed directly to the seabed means that the potential impact pathways for marine species and habitats may change. Although there may be reduced impacts in terms of noise, our knowledge of the environment and species distributions tends to decrease further offshore and in deeper water.

#### Data requirements

The environmental assessment process for offshore wind farms in Europe has highlighted the need for more synoptic studies to complement the site-specific surveys and monitoring that may be required around particular developments. Experience in Denmark, Germany and the Netherlands has highlighted the value of having a few key demonstrator sites to study interactions with key receptor species in these shallow North Sea areas e.g. [9,48,78]. Development of a broader suite of demonstrator sites is now required to understand potential interactions with a wider range of species and habitats that will result from the expansion of this industry. Such demonstrator sites could be focused on areas that build on existing research programs or where there are specific species of concern so that parameters of interest can be determined and models for assessing impacts developed and tested. Where regulators are required to consider potential population level effects on protected species, demonstrator sites should be selected to maximize the opportunity for linkage with individual based demographic studies [105]. This approach offers the potential to explore whether individual and colony specific variation in exposure to stressors such as noise or collision risk influences reproduction and survival rates. Critically, individual based studies can also be used to assess how the impacts of particular stressors interact with broader scale variation in environmental conditions e.g. [106] or vary over time e.g. [107]. For example, long-term data collection on bottlenose dolphins and harbor seals in the Moray Firth, Northeast Scotland, means that data on demography and fecundity are available as a baseline and can be used to determine if there are any changes in these vital rates during construction activities [58,108]. Focused studies such as these will be especially important for developing and testing the modeling frameworks that have been used to assess the impacts of construction noise [58] or collision risk [65], supporting a more general understanding of the longer-term population consequence of the short-term interactions recognized in current assessments. Information on these ecological processes that can only be obtained from a few focused studies at demonstrator sites can then be integrated with site specific data on distribution and abundance at proposed wind energy sites. This will provide more robust assessments of the population consequences of future developments.

## Conclusions

As offshore wind farms grow in size and number around the world, several changes in the priorities for environmental research and assessments are occurring. Firstly, there are an increasing number of cases where more than one wind farm project may occur within the home range of a population. Consequently, cumulative impact assessments, which should be made at the population level, will become increasingly important when assessing the effect of these activities on marine species and populations. Secondly, for species such as marine mammals, it is becoming increasingly clear that the most significant consequences of offshore wind farm construction and operation are likely to occur as a result of avoidance of construction noise or structures rather than direct mortality. Hence there needs to be a greater focus on assessing the longer-term impact of any behavioral responses through changes in energetic costs, survival or fecundity. Finally, as offshore wind farms increase in scale, there is a need to put any observed biological impacts into a population context. This requires an understanding of the relative scale of any impacts in relation to existing natural variation and other anthropogenic drivers such as fisheries bycatch or exploitation. Only then can the population consequences be modeled and conservation priorities be identified.

Drewitt and Langston [109] previously recommended a number of best practice measures for reducing the impacts of wind farms on birds. These recommendations included ensuring that key areas of conservation importance and sensitivity are avoided, grouping turbines to avoid alignment perpendicular to main flight paths or migration corridors, timing construction to avoid sensitive periods, and timing and routing maintenance trips to reduce disturbance from boats, helicopters and personnel. In practice, it is unlikely that all of these recommendations can be met given the challenge of balancing the needs of all stakeholders in the marine spatial planning process. In particular, many of the sites suitable for offshore wind energy development, such as offshore sandbanks, are also important habitats for marine species and fisheries. There is therefore a need for careful consideration in finer scale spatial planning and the identification of other mitigation measures to minimize environmental and human user conflicts. Strategic and targeted research around the next generation of offshore wind farm sites is now required to support the regulators’ need to achieve a balance between climate change targets and existing environmental legislation.

## Competing interests

The authors declare that they have no competing interests.

## Authors’ contributions

The manuscript was drafted by HB and revised and finalized together with KB and PT. All authors have read and approved the final manuscript.

## References

[B1] Toke D The UK offshore wind power programme: a sea-change in UK energy policy? Energy Policy 2011 39 526 534 10.1016/j.enpol.2010.08.043

[B2] Breton SP Moe G Status, plans and technologies for offshore wind turbines in Europe and North America Renew Energy 2009 34 646 654 10.1016/j.renene.2008.05.040

[B3] European Wind Energy Association The European Offshore Wind Industry - Key Trends and Statistics 2013 2014 Brussels, Belgium: A report by the European Wind Energy Association

[B4] Sun X Huang D Wu G The current state of offshore wind energy technology development Energy 2012 41 298 312 10.1016/j.energy.2012.02.054

[B5] Inger R Attrill MJ Bearhop S Broderick AC Grecian WJ Hodgson DJ Mills C Sheehan E Votier SC Witt MJ Godley BJ Marine renewable energy: potential benefits to biodiversity? An urgent call for research J Appl Ecol 2009 46 1145 1153

[B6] Boehlert GW Gill AB Environmental and ecological effects of ocean renewable energy development: a current synthesis Oceanography 2010 23 68 81 10.5670/oceanog.2010.46

[B7] Gill AB Offshore renewable energy: ecological implications of generating electricity in the coastal zone J Appl Ecol 2005 42 605 615 10.1111/j.1365-2664.2005.01060.x

[B8] Wilhelmsson D Malm T Öhman MC The influence of offshore windpower on demersal fish ICES J Mar Sci 2006 63 775 784 10.1016/j.icesjms.2006.02.001

[B9] Lindeboom HJ Kouwenhoven HJ Bergman MJN Bouma S Brasseur S Daan R Fijn RC De Haan D Dirksen S van Hal R Hille Ris Lambers R ter Hofstede R Krijgsveld KL Leopold M Scheidat M Short-term ecological effects of an offshore wind farm in the Dutch coastal zone; a compilation Environ Res Lett 2011 6 035101 10.1088/1748-9326/6/3/035101

[B10] Maar M Bolding K Petersen JK Hansen JLS Timmermann K Local effects of blue mussels around turbine foundations in an ecosystem model of Nysted off-shore wind farm, Denmark J Sea Res 2009 62 159 174 10.1016/j.seares.2009.01.008

[B11] Russell DJF Brasseur SMJM Thompson D Hastie GD Janik VM Aarts G McClintock BT Matthiopoulos J Moss SEW McConnell B Marine mammals trace anthropogenic structures at sea Curr Biol 2014 24 R638 R639 10.1016/j.cub.2014.06.033 25050956

[B12] Buck BH Krause G Rosenthal H Extensive open ocean aquaculture development within wind farms in Germany: the prospect of offshore co-management and legal constraints Ocean Coast Manag 2004 47 95 122 10.1016/j.ocecoaman.2004.04.002

[B13] Renewable UK Cumulative impact assessment guidelines: Guiding principles for cumulative impacts assessment in offshore wind farms 2013 Available at: http://www.renewableuk.com/en/publications/index.cfm/cumulative-impact-assessment-guidelines

[B14] Masden EA Fox AD Furness RW Bullman R Haydon DT Cumulative impact assessments and bird/wind farm interactions: Developing a conceptual framework Environ Impact Assess Rev 2010 30 1 7 10.1016/j.eiar.2009.05.002

[B15] Dolman S Simmonds M Towards best environmental practice for cetacean conservation in developing Scotland's marine renewable energy Mar Policy 2010 34 1021 1027 10.1016/j.marpol.2010.02.009

[B16] Madsen PT Wahlberg M Tougaard J Lucke K Tyack P Wind turbine underwater noise and marine mammals: implications of current knowledge and data needs Mar Ecol Prog Ser 2006 309 279 295

[B17] Thomsen F Lüdemann K Kafemann R Piper W Effects of offshore wind farm noise on marine mammals and fish 2006 Biola, Hamburg: Germany on behalf of COWRIE Ltd.

[B18] Popper AN Hastings MC The effects of human-generated sound on fish Integr Zool 2009 4 43 52 10.1111/j.1749-4877.2008.00134.x 21392276

[B19] Gill AB Bartlett M Thomsen F Potential interactions between diadromous fishes of U.K. conservation importance and the electromagnetic fields and subsea noise from marine renewable energy developments J Fish Biol 2012 81 664 695 10.1111/j.1095-8649.2012.03374.x 22803729

[B20] Popper AN Hastings MC The effects of anthropogenic sources of sound on fishes J Fish Biol 2009 75 455 489 10.1111/j.1095-8649.2009.02319.x 20738551

[B21] Tougaard J Henriksen OD Miller LA Underwater noise from three types of offshore wind turbines: Estimation of impact zones for harbor porpoises and harbor seals J Acoust Soc Am 2009 125 3766 3773 10.1121/1.3117444 19507958

[B22] Marmo B Roberts I Buckingham MP King S Booth C Modelling of noise effects of operational offshore wind turbines including noise transmission through various foundation types 2013 Edinburgh: Scottish Government

[B23] Desholm M Kahlert J Avian collision risk at an offshore wind farm Biol Lett 2005 1 296 298 10.1098/rsbl.2005.0336 17148191 PMC1617151

[B24] Furness RW Wade HM Masden EA Assessing vulnerability of marine bird populations to offshore wind farms J Environ Manag 2013 119 56 66 10.1016/j.jenvman.2013.01.025 23454414

[B25] Tricas T Gill A Normandeau, Exponent Effects of EMFs from undersea power cables on elasmobranchs and other marine species 2011 Camarillo, CA: U.S. Dept. of the Interior, Bureau of Ocean Energy Management, Regulation, and Enforcement, Pacific OCS Region OCS Study BOEMRE 2011–09

[B26] Gill AB Huang Y Gloyne-Philips I Metcalfe J Quayle V Spencer J Wearmouth V COWRIE 2.0 Electromagnetic Fields (EMF) Phase 2: EMF-sensitive fish response to EM emmisions from sub-sea electricity cables of the type used by the offshore renewable energy industry 2009 Thetford, UK: Commissioned by COWRIE Ltd (project reference COWRIE-EMF-1-06)

[B27] Westerberg H Lagenfelt I Sub-sea power cables and the migration behaviour of the European eel Fish Manag Ecol 2008 15 369 375 10.1111/j.1365-2400.2008.00630.x

[B28] Southall BL Bowles AE Ellison WT Finneran JJ Gentry RL Greene CR Jr Kastak D Ketten DR Miler JH Nachtigall PE Richardson WJ Thomas JA Tyack PL Marine mammal noise exposure criteria: initial scientific recommendations Aquat Mamm 2007 33 411 521 10.1578/AM.33.4.2007.411

[B29] National Research Council Marine Mammal Populations and Ocean Noise: Determining When Ocean Noise Causes Biologically Significant Effects 2005 Washington, DC: National Academy Press

[B30] Wahlberg M Westerberg H Hearing in fish and their reactions to sounds from offshore wind farms Mar Ecol Prog Ser 2005 288 295 309

[B31] Hawkins AD Popper AN Assessing the impacts of underwater sounds on fishes and other forms of marine life Acoust Today 2014 10 30 41 10.1121/1.4870174

[B32] Casper BM Halvorsen MB Matthews F Carlson TJ Popper AN Recovery of barotrauma injuries resulting from exposure to pile driving sound in two sizes of hybrid striped bass PLoS ONE 2013 8 e73844 10.1371/journal.pone.0073844 24040089 PMC3770664

[B33] Casper BM Popper AN Matthews F Carlson TJ Halvorsen MB Recovery of barotrauma injuries in Chinook salmon, *Oncorhynchus tshawytscha* from exposure to pile driving sound PLoS ONE 2012 7 e39593 10.1371/journal.pone.0039593 22745794 PMC3382140

[B34] Halvorsen MB Casper BM Matthews F Carlson TJ Popper AN Effects of exposure to pile-driving sounds on the lake sturgeon, Nile tilapia and hogchoker Proc R Soc Lond B Biol Sci 2012 279 4705 4714 10.1098/rspb.2012.1544 PMC3497083 23055066

[B35] Bolle LJ de Jong CAF Bierman SM Van Beek PJG Van Keeken OA Wessels PW Van Damme CJG Winter HV De Haan D Dekeling RPA Common sole larvae survive high levels of pile-driving sound in controlled exposure experiments PLoS ONE 2012 7 e33052 10.1371/journal.pone.0033052 22431996 PMC3303794

[B36] Thomsen F Mueller-Blenkle C Gill A Metcalfe J McGregor PK Bendall V Andersson MH Sigray P Wood D Popper AN, Hawkins A Effects of pile driving on the behavior of cod and sole The Effects of Noise on Aquatic Life Advances in Experimental Medicine and Biology, Volume 730 2012 New York, USA: Springer 387 388 10.1007/978-1-4419-7311-5_88 22278525

[B37] Mueller-Blenkle C Gill AB McGregor PK Andersson MH Sigray P Bendall V Metcalfe J Thomsen F Popper AN, Hawkins A A novel field study setup to investigate the behavior of fish related to sound The Effects of Noise on Aquatic Life Advances in Experimental Medicine and Biology, Volume 730 2012 New York, USA: Springer 389 391 10.1007/978-1-4419-7311-5_89 22278526

[B38] Kaiser MJ Galanidi M Showler DA Elliott AJ Caldow RWG Rees EIS Stillman RA Sutherland WJ Distribution and behaviour of Common Scoter Melanitta nigra relative to prey resources and environmental parameters Ibis 2006 s1 110 128

[B39] Pelletier SK Omland K Watrous KS Peterson TS Information Synthesis on the Potential for Bat Interactions with Offshore Wind Facilities - Final Report 2013 Herndon, VA: U.S. Department of the Interior, Bureau of Ocean Energy Management, Headquarters OCS Study BOEM 2013–01163

[B40] Sjollema AL Gates JE Hilderbrand RH Sherwell J Offshore activity of bats along the Mid-Atlantic Coast Northeast Nat 2014 21 154 163 10.1656/045.021.0201

[B41] Kunz TH Arnett EB Erickson WP Hoar AR Johnson GD Larkin RP Strickland MD Thresher RW Tuttle MD Ecological impacts of wind energy development on bats: questions, research needs, and hypotheses Front Ecol Environ 2007 5 315 324 10.1890/1540-9295(2007)5[315:EIOWED]2.0.CO;2

[B42] Waring GT Wood SA Josephson E Literature search and data synthesis for marine mammals and sea turtles in the U.S. Atlantic from Maine to the Florida Keys 2012 New Orleans, LA: U.S Department of the Interior, Bureau of Ocean Energy Management, Gulf of Mexico OCS Region OCS Study BOEM 2012–109

[B43] Dow Piniak WE Eckert SA Harms CA Stringer EM Underwater Hearing Sensitivity of The Leatherback Sea Turtle (Dermochelys coriacea): Assessing The Potential Effect of Anthropogenic Noise 2012 Herndon, VA: U.S Department of the Interior, Bureau of Ocean Energy Management, Headquarters OCS Study BOEM 2012–01156

[B44] Rein CG Lundin AS Wilson SJK Kimbrell E Offshore Wind Energy Development Site Assessment and Characterization: Evaluation of the Current Status and European Experience 2013 Herndon, VA: U.S. Department of the Interior, Bureau of Ocean Energy Management, Office of Renewable Energy Programs OCS Study BOEM 2013–0010

[B45] Carstensen J Henriksen OD Teilmann J Impacts of offshore wind farm construction on harbour porpoises: acoustic monitoring of echolocation activity using porpoise detectors (T-PODs) Mar Ecol Prog Ser 2006 321 295 308

[B46] Tougaard J Carstensen J Teilmann J Pile driving zone of responsiveness extends beyond 20 km for harbor porpoises (*Phocoena phocoena* (L.)) J Acoust Soc Am 2009 126 11 14 10.1121/1.3132523 19603857

[B47] Bailey H Senior B Simmons D Rusin J Picken G Thompson PM Assessing underwater noise levels during pile-driving at an offshore windfarm and its potential impact on marine mammals Mar Pollut Bull 2010 60 888 897 10.1016/j.marpolbul.2010.01.003 20152995

[B48] Brandt MJ Diederichs A Betke K Nehls G Responses of harbour porpoises to pile driving at the Horns Rev II offshore wind farm in the Danish North Sea Mar Ecol Prog Ser 2011 421 205 216

[B49] Thompson PM Lusseau D Barton T Simmons D Rusin J Bailey H Assessing the responses of coastal cetaceans to the construction of offshore wind turbines Mar Pollut Bull 2010 60 1200 1208 10.1016/j.marpolbul.2010.03.030 20413133

[B50] Kastelein RA Van Heerden D Gransier R Hoek L Behavioral responses of a harbor porpoise (*Phocoena phocoena*) to playbacks of broadband pile driving sounds Mar Environ Res 2013 92 206 214 24144856 10.1016/j.marenvres.2013.09.020

[B51] Koschinski S Culik BM Henriksen OD Tregenza N Ellis G Jansen C Kathe G Behavioural reactions of free-ranging porpoises and seals to the noise of a simulated 2 MW windpower generator Mar Ecol Prog Ser 2003 265 263 273

[B52] Degraer S Brabant R Rumes B Environmental impacts of offshore wind farms in the Belgian part of the North Sea: Learning from the past to optimise future monitoring programmes: 26–28 November 2013 2013 Brussels, Belgium: Royal Belgian Institute of Natural Sciences

[B53] Ellison WT Southall BL Clark CW Frankel AS A new context-based approach to assess marine mammal behavioral responses to anthropogenic sounds Conserv Biol 2012 26 21 28 10.1111/j.1523-1739.2011.01803.x 22182143

[B54] Diederichs A Nehls G Dähne M Adler S Koschinski S Verfuß U Methodologies for measuring and assessing potential changes in marine mammal behaviour, abundance or distribution arising from the construction, operation and decommissioning of offshore windfarms 2008 Germany: BioConsult SH report to COWRIE Ltd

[B55] Thompson PM Brookes KL Graham IM Barton TR Needham K Bradbury G Merchant ND Short-term disturbance by a commercial two-dimensional seismic survey does not lead to long-term displacement of harbour porpoises Proc R Soc Lond B Biol Sci 2013 280 20132001 10.1098/rspb.2013.2001 PMC3790491 24089338

[B56] Bailey H Hammond PS Thompson PM Modelling harbour seal habitat by combining data from multiple tracking systems J Exp Mar Biol Ecol 2014 450 30 39

[B57] Bailey H Clay G Coates EA Lusseau D Senior B Thompson PM Using T-PODs to assess variations in the occurrence of coastal bottlenose dolphins and harbour porpoises Aquat Conserv Mar Freshwat Ecosyst 2010 20 150 158 10.1002/aqc.1060

[B58] Thompson PM Hastie GD Nedwell J Barham R Brookes KL Cordes LS Bailey H McLean N Framework for assessing impacts of pile-driving noise from offshore wind farm construction on a harbour seal population Environ Impact Assess Rev 2013 43 73 85

[B59] Barrios L Rodríguez A Behavioural and environmental correlates of soaring-bird mortality at on-shore wind turbines J Appl Ecol 2004 41 72 81 10.1111/j.1365-2664.2004.00876.x

[B60] Garthe S Hüppop O Scaling possible adverse effects of marine wind farms on seabirds: developing and applying a vulnerability index J Appl Ecol 2004 41 724 734 10.1111/j.0021-8901.2004.00918.x

[B61] Camphuysen KCJ Fox TAD Leopold MMF Petersen IK Towards standardised seabirds at sea census techniques in connection with environmental impact assessments for offshore wind farms in the U Report by Royal Netherlands Institute for Sea Research and the Danish National Environmental Research Institute to COWRIE BAM 02–2002 2004 London: Crown Estate Commissioners

[B62] Cook ASCP Johnston A Wright LJ Burton NHK A Review of Flight Heights and Avoidance Rates of Birds in Relation to Offshore Wind Farms 2012 Norfolk, UK: British Trust for Ornithology on behalf of The Crown Estate, Project SOSS-02 BTO Research Report Number 618

[B63] Johnston A Cook ASCP Wright LJ Humphreys EM Burton NHK Modelling flight heights of marine birds to more accurately assess collision risk with offshore wind turbines J Appl Ecol 2014 51 31 41 10.1111/1365-2664.12191

[B64] Plonczkier P Simms IC Radar monitoring of migrating pink-footed geese: behavioural responses to offshore wind farm development J Appl Ecol 2012 49 1187 1194 10.1111/j.1365-2664.2012.02181.x

[B65] Chamberlain DE Rehfisch MR Fox AD Desholm M Anthony SJ The effect of avoidance rates on bird mortality predictions made by wind turbine collision risk models Ibis 2006 148 198 202

[B66] Masden EA Haydon DT Fox AD Furness RW Barriers to movement: Modelling energetic costs of avoiding marine wind farms amongst breeding seabirds Mar Pollut Bull 2010 60 1085 1091 10.1016/j.marpolbul.2010.01.016 20188382

[B67] Busch M Kannen A Garthe S Jessopp M Consequences of a cumulative perspective on marine environmental impacts: Offshore wind farming and seabirds at North Sea scale in context of the EU Marine Strategy Framework Directive Ocean Coast Manag 2013 71 213 224

[B68] McCann J Developing Environmental Protocols and Modeling Tools to Support Ocean Renewable Energy and Stewardship 2012 Herndon, VA: U.S. Department of the Interior, Bureau of Ocean Energy Management, Office of Renewable Energy Programs OCS Study BOEM 2012–082

[B69] Teilmann J Carstensen J Negative long term effects on harbour porpoises from a large scale offshore wind farm in the Baltic - evidence of slow recovery Environ Res Lett 2012 7 045101 10.1088/1748-9326/7/4/045101

[B70] Wade PR Calculating limits to the allowable human-caused mortality of cetaceans and pinnipeds Mar Mammal Sci 1998 14 1 37 10.1111/j.1748-7692.1998.tb00688.x

[B71] Butler JRA Middlemas SJ McKelvey SA McMyn I Leyshon B Walker I Thompson PM Boyd IL Duck C Armstrong JD Graham IM Baxter JM The Moray Firth Seal Management Plan: an adaptive framework for balancing the conservation of seals, salmon, fisheries and wildlife tourism in the UK Aquat Conserv Mar Freshwat Ecosyst 2008 18 1025 1038 10.1002/aqc.923

[B72] New LF Clark JS Costa DP Fleishman E Hindell MA Klanjšček T Lusseau D Kraus S McMahon CR Robinson PW Schick RS Schwarz LK Simmons SE Thomas L Tyack P Harwood J Using short-term measures of behaviour to estimate long-term fitness of southern elephant seals Mar Ecol Prog Ser 2014 496 99 108

[B73] Harwood J King S Schick R Donovan C Booth C A protocol for implementing the interim population consequences of disturbance (PCoD) approach: Quantifying and assessing the effects of UK offshore renewable energy developmenets on marine mammal populations: Report number SMRUL-TCE-2013-014 Scott Mar Freshwater Sci 2014 5 2

[B74] Frederiksen M Wanless S Harris MP Rothery P Wilson LJ The role of industrial fisheries and oceanographic change in the decline of North Sea black-legged kittiwakes J Appl Ecol 2004 41 1129 1139 10.1111/j.0021-8901.2004.00966.x

[B75] Underwood AJ On beyond BACI: Sampling designs that might reliably detect environmental disturbances Ecol Appl 1994 4 3 15 10.2307/1942110

[B76] Hewitt JE Thrush SE Cummings VJ Assessing environmental impacts: Effects of spatial and temporal variability at likely impact scales Ecol Appl 2001 11 1502 1516 10.1890/1051-0761(2001)011[1502:AEIEOS]2.0.CO;2

[B77] Ellis JI Schneider DC Evaluation of a gradient sampling design for environmental impact assessment Environ Monit Assess 1997 48 157 172 10.1023/A:1005752603707

[B78] Dähne M Gilles A Lucke K Peschko V Adler S Krügel K Sundermeyer J Siebert U Effects of pile-driving on harbour porpoises (*Phocoena phocoena*) at the first offshore wind farm in Germany Environ Res Lett 2013 8 025002 10.1088/1748-9326/8/2/025002

[B79] Heupel MR Semmens JM Hobday AJ Automated acoustic tracking of aquatic animals: scales, design and deployment of listening station arrays Mar Freshw Res 2006 57 1 13 10.1071/MF05091

[B80] Scales KL Lewis JA Lewis JP Castellanos D Godley BJ Graham RT Insights into habitat utilisation of the hawksbill turtle, *Eretmochelys imbricata* (Linnaeus, 1766), using acoustic telemetry J Exp Mar Biol Ecol 2011 407 122 129 10.1016/j.jembe.2011.07.008

[B81] Espinoza M Farrugia TJ Webber DM Smith F Lowe CG Testing a new acoustic telemetry technique to quantify long-term, fine-scale movements of aquatic animals Fish Res 2011 108 364 371 10.1016/j.fishres.2011.01.011

[B82] Maxwell SM Hazen EL Bograd SJ Halpern BS Breed GA Nickel B Teutschel NM Crowder LB Benson S Dutton PH Bailey H Kappes MA Kuhn CE Weise MJ Mate B Shaffer SA Hassrick JL Henry RW Irvine L McDonald BI Robinson PW Block BA Costa DP Cumulative human impacts on marine predators Nat Commun 2013 4 2688 24162104 10.1038/ncomms3688

[B83] Punt MJ Groeneveld RA Van Ierland EC Stel JH Spatial planning of offshore wind farms: A windfall to marine environmental protection? Ecol Econ 2009 69 93 103 10.1016/j.ecolecon.2009.07.013

[B84] Band W Madders M Whitfield DP De Lucas M, Janss G, Ferrer M Developing field and analytical methods to assess avian collision risk at wind farms Birds and Wind Power 2005 Barcelona, Spain: Lynx Edicions

[B85] Band B Band B Using a collision risk model to assess bird collision risks for offshore windfarms 2012 Norway: SOSS report for The Crown Estate

[B86] Gordon J Gillespie D Potter J Frantzis A Simmonds MP Swift R Thompson D A review of the effects of seismic surveys on marine mammals Mar Technol Soc J 2003 37 16 34 10.4031/002533203787536998

[B87] OSPAR Overview of the impacts of anthropogenic underwater sound in the marine environment 2009 North-East Atlantic: OSPAR Convention for the Protection of the Marine Environment of the North-East Atlantic http://www.ospar.org

[B88] Nedwell JR Parvin SJ Edwards B Workman R Brooker AG Kynoch JE Nedwell JR Parvin SJ Edwards B Workman R Brooker AG Kynoch JE Measurement and interpretation of underwater noise during construction and operation of offshore windfarms in UK waters Subacoustech Report No. 544R0738 to COWRIE Ltd 2007 978-0-9554279-5-4

[B89] Di Iorio L Clark CW Exposure to seismic survey alters blue whale acoustic communication Biol Lett 2010 6 51 54 10.1098/rsbl.2009.0651 19776059 PMC2817268

[B90] Castellote M Clark CW Lammers MO Acoustic and behavioural changes by fin whales (*Balaenoptera physalus*) in response to shipping and airgun noise Biol Conserv 2012 147 115 122 10.1016/j.biocon.2011.12.021

[B91] Blackwell SB Nations CS McDonald TL Greene CR Thode AM Guerra M Macrander AM Effects of airgun sounds on bowhead whale calling rates in the Alaskan Beaufort Sea Mar Mammal Sci 2013 29 E342 E365

[B92] Harris RE Miller GW Richardson WJ Seal responses to airgun sounds during summer seismic surveys in the Alaskan Beaufort Sea Mar Mammal Sci 2001 17 795 812 10.1111/j.1748-7692.2001.tb01299.x

[B93] DeRuiter SL Doukara KL Loggerhead turtles dive in response to airgun sound exposure Endanger Species Res 2012 16 55 63 10.3354/esr00396

[B94] Rutenko AN Borisov SV Gritsenko AV Jenkerson MR Calibrating and monitoring the western gray whale mitigation zone and estimating acoustic transmission during a 3D seismic survey, Sakhalin Island, Russia Environ Monit Assess 2007 134 21 44 10.1007/s10661-007-9814-z 17762974 PMC2798049

[B95] Nowacek DP Vedenev A Southall BL Racca R Popper AN, Hawkins A Development and implementation of criteria for exposure of western gray whales to oil and gas industry noise The Effects of Noise on Aquatic Life Advances in Experimental Medicine and Biology, Volume 730 2012 New York, USA: Springer 523 528 10.1007/978-1-4419-7311-5_119 22278556

[B96] Weir CR Short-finned pilot whales (*Globicephala macrorhynchus*) respond to an airgun ramp-up procedure off Gabon Aquat Mamm 2008 34 349 354 10.1578/AM.34.3.2008.349

[B97] JNCC Statutory Nature Conservation Agency Protocol for Minimising the Risk of Injury to Marine Mammals from Piling Noise 2010 Aberdeen, UK: Joint Nature Conservation Committee

[B98] Parsons ECM Dolman SJ Jasny M Rose NA Simmonds MP Wright AJ A critique of the UK’s JNCC seismic survey guidelines for minimising acoustic disturbance to marine mammals: Best practise? Mar Pollut Bull 2009 58 643 651 10.1016/j.marpolbul.2009.02.024 19342066

[B99] Miller PJO Johnson MP Madsen PT Biassoni N Quero M Tyack PL Using at-sea experiments to study the effects of airguns on the foraging behavior of sperm whales in the Gulf of Mexico Deep-Sea Res I 2009 56 1168 1181 10.1016/j.dsr.2009.02.008

[B100] Van Parijs SM Clark CW Sousa-Lima RS Parks SE Rankin S Risch D Van Opzeeland IC Management and research applications of real-time and archival passive acoustic sensors over varying temporal and spatial scales Mar Ecol Prog Ser 2009 395 21 36

[B101] Bellmann MA Remmers P Noise mitigation systems (NMS) for reducing pile driving noise: Experiences with the “big bubble curtain” relating to noise reduction J Acoust Soc Am 2013 134 4059

[B102] European Wind Energy Association Deep Water: The Next Step for Offshore Wind Energy 2013 Brussels, Belgium: A report by the European Wind Energy Association

[B103] European Wind Energy Association The European Offshore Wind Industry Key 2011 Trends and Statistics 2012 Brussels, Belgium: A report by the European Wind Energy Association

[B104] Stokes I Hotspots: Scotland and Fukushima Renewable Energy Focus 2013 14 10 11

[B105] Clutton-Brock T Sheldon BC Individuals and populations: the role of long-term, individual-based studies of animals in ecology and evolutionary biology Trends Ecol Evol 2010 25 562 573 10.1016/j.tree.2010.08.002 20828863

[B106] Votier SC Hatchwell BJ Beckerman A McCleery RH Hunter FM Pellatt J Trinder M Birkhead TR Oil pollution and climate have wide-scale impacts on seabird demographics Ecol Lett 2005 8 1157 1164 10.1111/j.1461-0248.2005.00818.x 21352439

[B107] Véran S Gimenez O Flint E Kendall WL Doherty PF Lebreton JD Quantifying the impact of longline fisheries on adult survival in the black-footed albatross J Appl Ecol 2007 44 942 952 10.1111/j.1365-2664.2007.01346.x

[B108] New LF Harwood J Thomas L Donovan C Clark JS Hastie G Thompson PM Cheney B Scott-Hayward L Lusseau D Modelling the biological significance of behavioural change in coastal bottlenose dolphins in response to disturbance Funct Ecol 2013 27 314 322 10.1111/1365-2435.12052

[B109] Drewitt AL Langston RHW Assessing the impacts of wind farms on birds Ibis 2006 148 29 42

